# Complete Surgical Resection of Rare Solitary Anterior Mediastinal Metastasis From Ovarian Clear Cell Carcinoma

**DOI:** 10.1002/rcr2.70809

**Published:** 2026-11-03

**Authors:** Teruhisa Chiba, Kaito Yano, Tomohiro Imoto, Mikito Suzuki, Reiko Shimizu, Tsunekazu Hishima, Kazuo Nakagawa

**Affiliations:** ^1^ Department of Thoracic Surgery Tokyo Metropolitan Cancer and Infectious Diseases Center Komagome Hospital Tokyo Japan; ^2^ Department of Pathology Tokyo Metropolitan Cancer and Infectious Diseases Center Komagome Hospital Tokyo Japan

**Keywords:** anterior mediastinum, clear cell carcinoma, hematogenous metastasis, ovarian cancer, surgical resection

## Abstract

Epithelial ovarian cancer typically spreads via transcoelomic dissemination and lymphatic pathways. Extra‐abdominal metastases presenting as solitary anterior mediastinal lesions are rare. Ovarian clear cell carcinoma (CCC) is characterized by aggressive local behaviour but has a lower propensity for distant metastasis than serous carcinoma. Furthermore, it is less sensitive to platinum‐based chemotherapy than other epithelial ovarian cancers. Here, we report a rare case of ovarian CCC with a solitary anterior mediastinal metastasis after primary surgery. A woman in her 50s had a 2.6‐cm anterior mediastinal mass during postoperative surveillance. Considering progressive lesion enlargement and increased 18F‐fluorodeoxyglucose uptake on positron emission tomography‐computed tomography, complete surgical resection via median sternotomy was performed. Histopathological and immunohistochemical findings confirmed the presence of metastatic ovarian CCC. The patient remained disease‐free for 6 months postoperatively. Complete surgical resection may represent an effective treatment option for isolated recurrence of chemoresistant histological subtypes.

## Graphical abstract

Here, we report an extremely rare case of a solitary anterior mediastinal metastasis from ovarian clear cell carcinoma. The lesion was successfully managed with complete R0 surgical resection via median sternotomy. In patients with a history of ovarian clear cell carcinoma, aggressive surgical intervention should be considered for isolated thoracic recurrences to overcome potential chemoresistance.

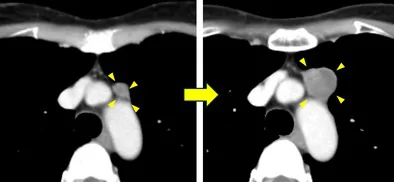

## 1 Introduction

The primary metastatic pathways of epithelial ovarian cancer include transcoelomic dissemination within the peritoneal cavity and lymphatic spread to the pelvic and para‐aortic lymph nodes [1]. Extra‐abdominal metastasis beyond the diaphragm is rare, and presentation as a solitary anterior mediastinal lesion is exceedingly rare. Ovarian clear cell carcinoma (CCC) exhibits more local invasiveness but a lower incidence of distant metastasis than serous carcinoma [2]. Here, we report a rare case of solitary anterior mediastinal metastasis from ovarian CCC following primary surgery that was successfully treated with complete surgical resection.

## 2 Case Report

A woman in her 50s underwent total hysterectomy, bilateral salpingo‐oophorectomy, partial omentectomy and pelvic lymphadenectomy for ovarian CCC (FIGO Stage IC3). Postoperatively, the patient received five cycles of adjuvant chemotherapy comprising paclitaxel and carboplatin (TC regimen). Two years postoperatively, follow up computed tomography (CT) revealed a small nodule in the anterior mediastinum (Figure 1A). The lesion gradually increased in size over 1 year, prompting referral to our department. Chest CT demonstrated a 2.6‐cm anterior mediastinal mass adjacent to the aortic arch, containing enhancing solid components and low‐attenuation areas, suggestive of necrosis or cystic degeneration (Figure 1B). Positron emission tomography (PET)‐CT revealed intense 18F‐fluorodeoxyglucose (FDG) uptake (maximum FDG uptake; 8.6, Figure 1C). No metastatic lesions were observed. The levels of tumour markers, including CEA (1.7 ng/mL) and CA125 (15.2 U/mL), were normal.

**FIGURE 1:**
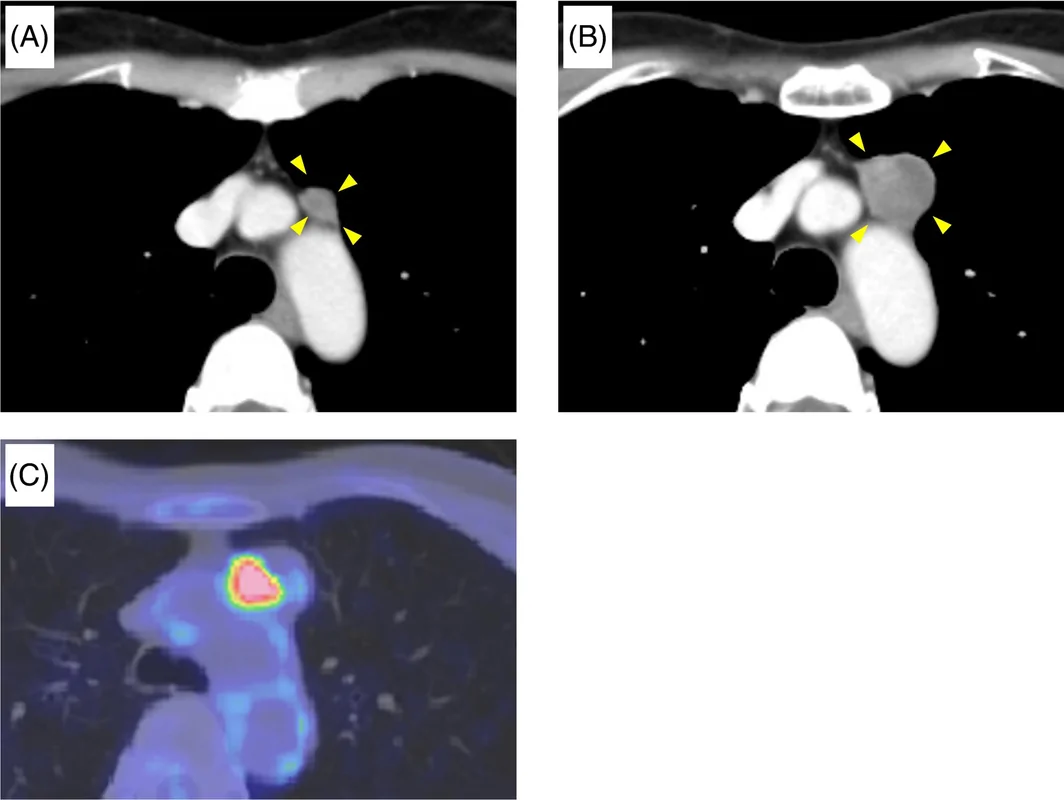
Radiological findings of the anterior mediastinal tumour. (A) Follow‐up chest CT performed 2 years after primary surgery reveals a small nodule in the anterior mediastinum (arrowheads). (B) One year later, contrast‐enhanced chest CT demonstrates enlargement of the lesion to 2.6 cm, showing enhancing solid components and low‐attenuation areas suggestive of necrosis or cystic degeneration (arrowheads). (C) PET‐CT demonstrates intense FDG uptake in the anterior mediastinal tumour with a maximum standardized uptake value of 8.6. CT, computed tomography; FDG, fluorodeoxyglucose; PET, positron emission tomography.

As differential diagnoses included cystic thymoma and solitary metastasis, surgical resection was performed for diagnostic and therapeutic purposes. As the lesion was adjacent to the aortic arch and invasion of the surrounding structures could not be excluded preoperatively, a median sternotomy approach was selected to facilitate complete resection. Intraoperatively, invasion into the left upper lobe of the lung and left phrenic nerve was observed. En bloc resection of the tumour, adjacent lung tissue and phrenic nerve was performed. The operative time was 168 min, and blood loss was 170 mL.

Histopathological examination revealed atypical cells with an abundant clear cytoplasm and hobnail features (Figure 2A–C). Morphological and immunohistochemical findings were consistent with metastatic ovarian CCC originating from the primary tumour (Figure 2D). The postoperative course was uneventful, and the patient was discharged on postoperative day six. Although adjuvant chemotherapy was considered, no additional treatments were administered at the patient's request. The patient has remained recurrence‐free for 6 months postoperatively.

**FIGURE 2:**
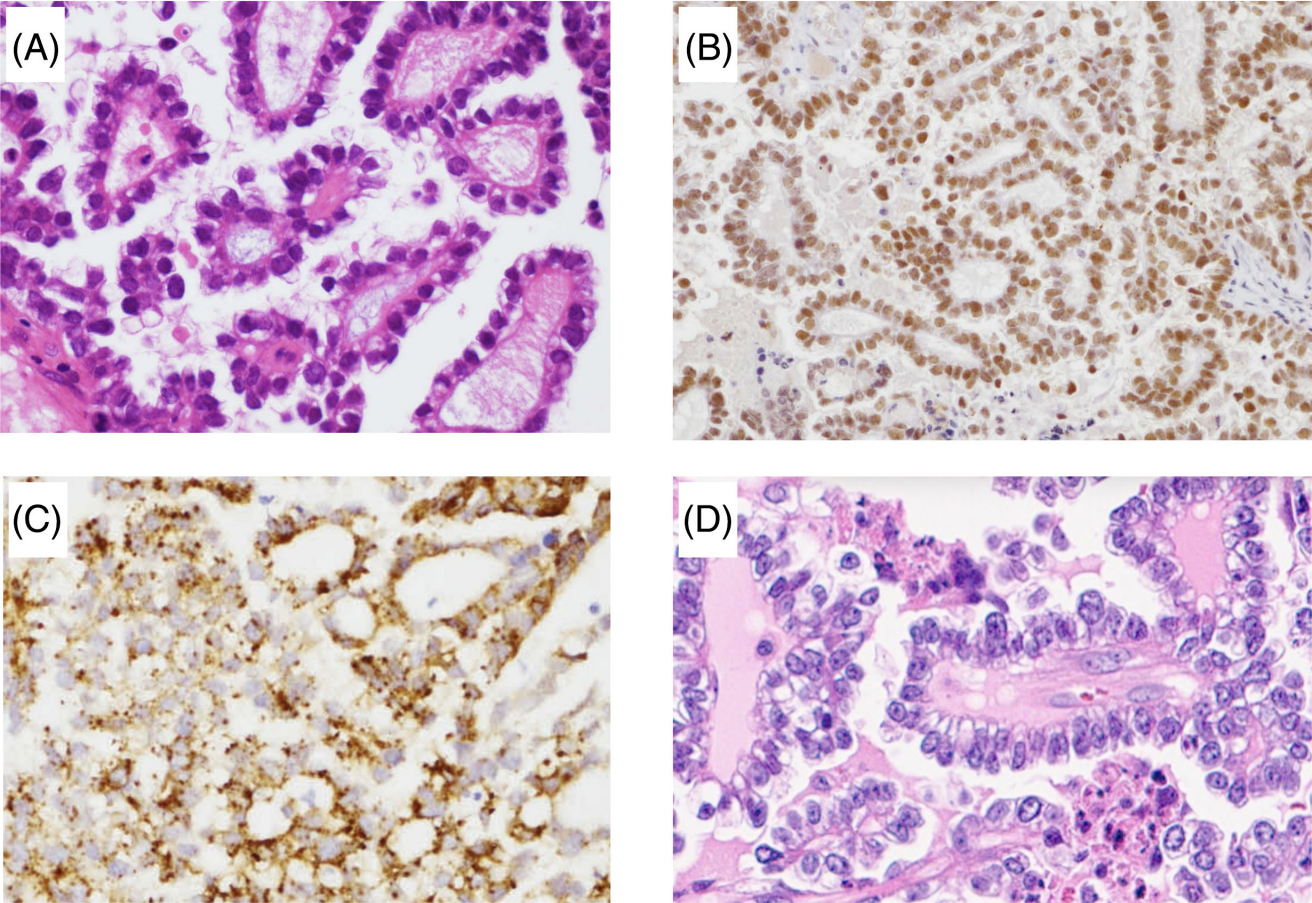
Histopathological and immunohistochemical findings of the mediastinal metastasis and primary ovarian tumour. (A) H&E staining shows papillary proliferation of cuboidal tumour cells with abundant clear cytoplasm and characteristic hobnail appearance (×200). (B) Immunohistochemical analysis reveals diffuse nuclear positivity for HNF‐1β. (C) Immunohistochemical analysis demonstrates strong cytoplasmic positivity for Napsin A. (D) H&E staining of the resected ovarian tumour demonstrates papillary proliferation of cuboidal tumour cells, interspersed with abundant clear cytoplasm and characteristic hobnail features (×200). H&E, haematoxylin and eosin.

## 3 Discussion

Extra‐abdominal metastases of ovarian cancer are most commonly observed in the pleura, lungs and supraclavicular lymph nodes; however, solitary anterior mediastinal metastases are exceedingly rare. To the best of our knowledge, only five cases of solitary anterior mediastinal metastasis from ovarian cancer (excluding lymph node metastases), including our case, have been reported (Table 1).

**TABLE 1:** Reported cases of solitary anterior mediastinal metastasis from ovarian cancer, including the present case.

First author	Histological type	Recurrence site	Interval to recurrence	Treatment	Outcome
Montero [3]	Low grade serous	Inferior retrosternum	Synchronous metastasis	Resection	NED (6 mo)
Inoue [4]	Papillary serous	Cardiophrenic	348 mo	Resection chemotherapy	NED (12 mo)
Omura [5]	Low grade serous	Thymus	372 mo	Resection	Recurrence in IVC LN and abdominal wall at 19 mo, treated with resection; NED at 48 mo.
Miura [6]	High grade Serous	Inferior retrosternum	192 mo	Resection chemotherapy radiotherapy	NED (10 mo)
Present case	Clear cell carcinoma	Superior retrosternum	24 mo	Resection	NED (6 mo)

Abbreviations: IVC, inferior vena cava; LN, lymph node; mo, month; NED, no evidence of disease.

The metastatic pathway warrants consideration because it may provide insights into the biological behaviour of this unusual pattern of recurrence. Ovarian cancer metastasizes to the thoracic cavity via lymphatic channels via the diaphragmatic stomata, reaching the parasternal and cardiophrenic lymph nodes [1]. Previously, tumours were often located near the diaphragm, suggesting possible direct extension through the diaphragmatic stomata. Contrastingly, the tumour was located in the upper to middle anterior mediastinum at the aortic arch level, which is anatomically distant from the diaphragm. No mediastinal or cardiophrenic lymph node enlargement was observed. Therefore, hematogenous spread was considered as a metastatic pathway.

Moreover, previous cases involved serous carcinoma; to the best of our knowledge, this is the first reported case of solitary anterior mediastinal metastasis from ovarian CCC. CCC is characterized by a higher propensity for local invasion and lower tendency for distant metastasis than serous carcinoma, which may explain the rarity of isolated anterior mediastinal metastases in this histological subtype.

Adjuvant chemotherapy for advanced ovarian cancer delays recurrence, with a median progression‐free survival of approximately 18 months [7]. Although local interventions offer potential benefits for oligorecurrence, optimal treatment strategies for oligometastasis in ovarian cancer remain unclear. Notably, for localized recurrence occurring ≥ 6 months after completing initial chemotherapy, achieving complete resection through secondary cytoreductive surgery prolongs survival [8]. CCC is less sensitive to platinum‐based chemotherapy than other epithelial ovarian cancers [2]. Therefore, in cases of isolated recurrence with potential chemoresistance, as in this case, achieving complete surgical resection may represent a valuable treatment option for local disease control, although surgery alone is unlikely to be curative.

In this case, the CT findings; mixed solid and low‐attenuation components, were nonspecific, making it difficult to distinguish them from primary mediastinal tumours [9]. However, an increase in the size of the anterior mediastinal tumour on follow‐up CT prompted surgical intervention for diagnostic and therapeutic purposes. In patients with a history of malignancy, metastatic disease should be considered if an anterior mediastinal mass is detected.

Overall, we report a rare case of a solitary anterior mediastinal metastasis, representing hematogenous spread, occurring 3 years postoperatively for ovarian CCC. Although this is an extremely uncommon pattern of recurrence, complete surgical resection may offer durable disease control in histological subtypes that are potentially resistant to chemotherapy. Clinicians should consider metastatic disease in the differential diagnosis of anterior mediastinal tumours in patients with a history of ovarian cancer, even for histological subtypes with low metastatic potential.

## Author Contributions

**Teruhisa Chiba:** conceptualization, data curation, project administration, visualization, writing – original draft, writing – review and editing. **Kaito Yano:** conceptualization, writing – original draft, writing – review and editing, visualization, project administration, data curation. **Tomohiro Imoto:** data curation, writing – review and editing. **Mikito Suzuki:** data curation, writing – review and editing. **Reiko Shimizu:** data curation, writing – review and editing. **Tsunekazu Hishima:** writing – review and editing. **Kazuo Nakagawa:** supervision, writing – review and editing.

## Funding

The authors have nothing to report.

## Ethics Statement

As this manuscript describes a single case report of standard clinical care, institutional review board approval was waived.

## Consent

The authors declare that written informed consent was obtained for the publication of this manuscript and the accompanying images using the consent form provided by the journal.

## Conflicts of Interest

The authors declare no conflicts of interest.

## Data Availability

Data sharing not applicable to this article as no datasets were generated or analysed during the current study.
